# Computational identification of candidate biomarkers and compounds associated with skeletal muscle insulin sensitivity improvement mediated by physical activity: an integrative multi-omics and computational chemistry study

**DOI:** 10.3389/fbinf.2026.1885995

**Published:** 2026-10-07

**Authors:** Hong Liu, Zicheng Liang, Jun Tan, Chunyun Yuan

**Affiliations:** 1 Department of Geriatrics, Hunan Provincial Hospital of Integrated Traditional Chinese and Western Medicine (The Affiliated Hospital of Hunan Academy of Traditional Chinese Medicine), Changsha, China; 2 Second Clinical Medical School, Zhejiang Chinese Medical University, Hangzhou, China; 3 Graduate School, Hunan University of Chinese Medicine, Changsha, China

**Keywords:** deep learning, DFT, insulin sensitivity, MM-GBSA, molecular dynamics simulation, skeletal muscle, WNT4

## Abstract

**Background:**

Insulin resistance (IR) underlies many chronic diseases. While physical activity improves skeletal muscle insulin sensitivity (IS), the underlying molecular mechanisms and targeted drug strategies remain elusive. This hypothesis-generating study integrates bioinformatics and computational chemistry using public datasets; all findings are computational predictions requiring experimental validation.

**Methods:**

Using a skeletal muscle dataset (GSE24215, n = 10) of bed rest-induced IR and exercise-reversed IR, candidate genes were identified via WGCNA and differential expression analysis. Machine learning, GSEA, and regulatory network construction identified key targets and predicted mechanisms. To address the lack of targeted drugs, a deep learning-driven drug discovery and multi-scale binding validation strategy screened candidate compounds from natural and marine product databases.

**Results:**

WNT4 was the only candidate gene repeatedly identified across both phases, displaying a bidirectional expression pattern of “upregulation after bed rest and downregulation after exercise retraining.” GSEA predicts WNT4 expression is positively correlated with energy metabolism pathways (e.g., oxidative phosphorylation) and is mediated by predicted transcriptional (centered on EGR1) and post-transcriptional (hsa-miR-1238-3p/lncRNAs) regulatory networks. From over 700,000 screened compounds, three top candidates were identified. Molecular dynamics, binding free energy calculations, and quantum chemical analysis preliminarily predicted their binding stability and electronic structure features.

**Conclusion:**

This study proposes that WNT4 may play a role in the dynamic regulation of skeletal muscle IS, presenting a functional hypothesis of “endogenous compensation–stage transition”. It establishes a computational drug discovery pipeline providing a candidate compound library for WNT4. Given the limited sample size (n = 10), all findings require further validation in independent cohorts and experiments.

## 1 Introduction

Insulin resistance (IR) is characterized by a decreased ability of insulin-targeted tissues to respond to normal levels of insulin, resulting in impaired glucose uptake and disturbed lipid metabolism (Lee et al., 2022; Saltiel, 2021). IR is a key pathologic basis for the development of a variety of chronic noncommunicable diseases (such as type 2 diabetes, metabolic syndrome, and cardiovascular diseases) (Adeva-Andany et al., 2019). A systematic review that included 86 epidemiologic studies of IR noted that the prevalence of IR in the adult population (≥18 years of age) was 26.53%, highlighting it as a serious public health problem (Ballena-Caicedo et al., 2025).

Current clinical interventional drugs primarily used to improve IR include biguanides, thiazolidinediones, and GLP-1 receptor agonists; however, their limited scope of application and certain adverse effects suggest an urgent clinical need for alternative therapies (Dahlén et al., 2022). Non-pharmacological intervention strategies, such as physical activity and nutritional support, have been adopted as foundational regimens for IR management due to their comprehensive benefits (Iaccarino et al., 2021; Mastrototaro and Roden, 2021). Among these, physical activity can modulate insulin sensitivity (IS) through skeletal muscle (Whytock and Goodpaster, 2025). Studies in non-obese IR populations have shown that exercise alters the body’s metabolic pattern, inducing a shift from glucose dependence to lipid dependence (Amaravadi et al., 2025). Skeletal muscle accounts for more than 80% of postprandial oral glucose disposal; it is not only a major tissue for maintaining glucose homeostasis but also participates in the regulation of systemic IS through the secretion of myokines (Whytock and Goodpaster, 2025; Kaura and Hopkins, 2024; Hingst et al., 2022; Sammut et al., 2025). Recent multi-omics studies have begun to map molecular networks associated with the beneficial effects of exercise (Khokhar et al., 2022a), and similar strategies have revealed regulatory networks in type 2 diabetes and obesity-related IR (Khokhar et al., 2022b; Roy et al., 2024). However, the dynamic mechanisms by which physical activity regulates skeletal muscle IS remain largely unknown, which limits target screening and translational applications to some extent (Whytock and Goodpaster, 2025).

To initially explore this issue, this study selected a skeletal muscle microarray dataset that includes bidirectional interventions of IR induction and exercise-mediated reversal (Alibegovic et al., 2010). This dataset allows for a systematic comparison of the dynamic changes in IS across different stages, providing ideal material for identifying reversible targets. Based on this bidirectional regulatory cohort, the present study integrates multiple bioinformatics strategies—such as weighted gene co-expression network analysis (WGCNA), differentially expressed gene (DEG) analysis, and multi-algorithm machine learning joint validation—to systematically and preliminarily mine reversible pivotal targets associated with IS. Furthermore, to address the bottlenecks of traditional target development, such as long lead times and poor druggability, we constructed a computational drug discovery system centered on virtual screening, locally deployed AI models, marine natural product databases, molecular dynamics simulations, and quantum chemical calculations. This method preliminarily screens a candidate compound library with excellent drug-like properties for the target candidates. It should be noted that this study is based on a public dataset with a limited sample size (GSE24215, n = 10). All findings are therefore computational predictions and require further validation in independent cohorts and experiments. The systematic workflow of the study design is illustrated in Figure 1.

**FIGURE 1:**
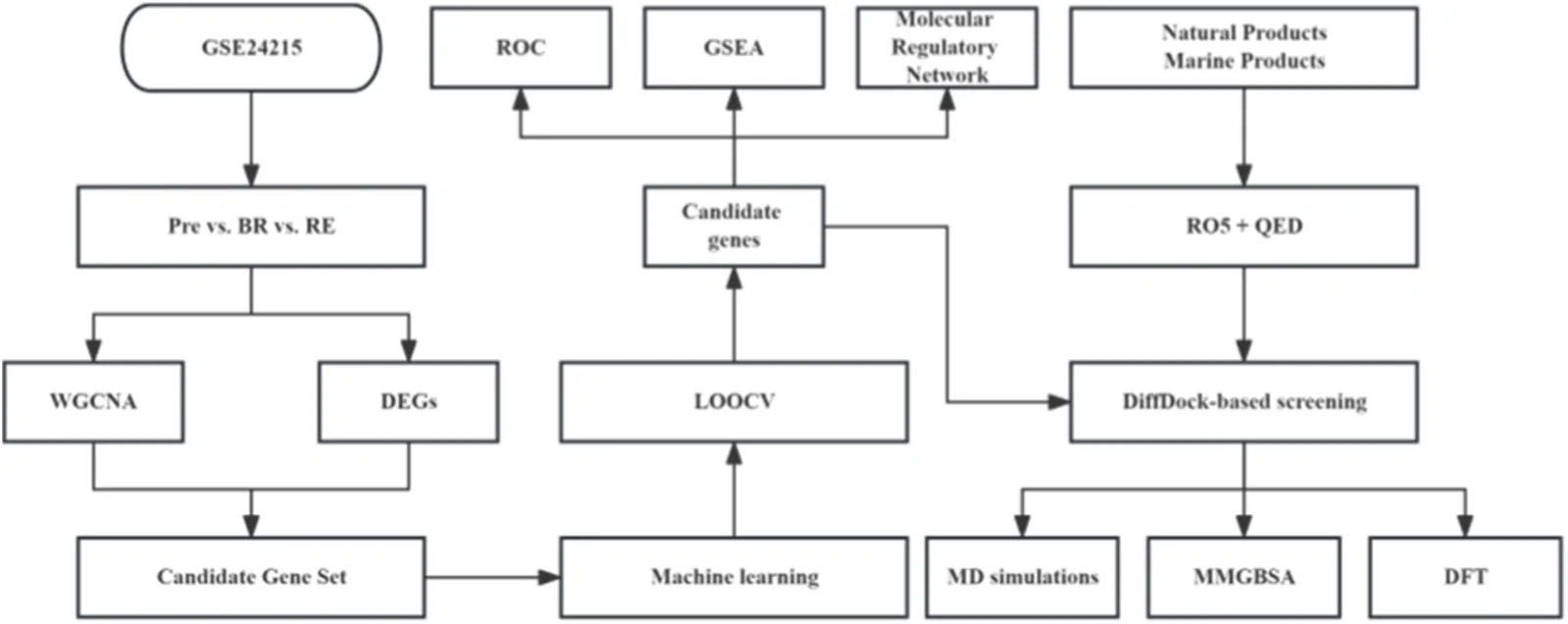
Study workflow.

## 2 Methods

### 2.1 Disease target discovery

#### 2.1.1 Data source

The dataset analyzed in this study was obtained from the GEO database (GSE24215, https://www.ncbi.nlm.nih.gov/geo/) (Barrett et al., 2013). This dataset contains samples of the vastus lateralis muscle from the same batch of 10 subjects across three phases: the initial baseline (Pre), after 9 days of bed rest (BR), and after 4 weeks of exercise retraining (RE), to compare the dynamic effects of physical activity on the IS.

#### 2.1.2 Candidate gene set screening

Gene co-expression network was constructed using the R package WGCNA (Liu Y. et al., 2023), and after eliminating the outlier samples, the optimal soft threshold β was determined using pickSoftThreshold to construct the topological overlap matrix (TOM), and finally the genes with similar expression patterns were assigned to different modules by average linkage hierarchical clustering to screen the key modules related to the phenotype. It should be noted that WGCNA is positioned as a preliminary exploratory screening tool in this study, and its candidate genes need to be further filtered through subsequent differentially expressed genes (DEGs) and machine learning consensus.

Meanwhile, the GEO2R tool (limma package) was used for intergroup normalization and differential expression analysis. The Benjamini-Hochberg (BH) method was applied for FDR multiple testing correction, with a corrected P-value <0.05 and |log_2_FC| > 1 as the threshold to identify DEGs. Given that this dataset has a repeated measures design, the differential expression analysis employed limma’s paired t-test framework to control for inter-individual variability. The above DEGs were intersected with the key module genes from WGCNA to obtain a candidate gene set for subsequent machine learning analysis.

#### 2.1.3 Screening and evaluation of candidate targets

To further screen reliable candidate targets from the candidate gene set, this study applied multiple machine learning algorithms: The “glmnet” package was used for least absolute shrinkage and selection operator (LASSO) regression analysis (random seed = 12,345), with 10-fold cross-validation to select the λ value (lambda.min) that minimizes the average cross-validation error as the penalty parameter, ensuring model sparsity and reducing the risk of overfitting (Engebretsen and Bohlin, 2019; Wu et al., 2023); the “randomForest” package was used to perform the random forest (RF) algorithm (random seed = 2025), constructing 5000 decision trees, with feature importance evaluated using the algorithm’s built-in out-of-bag (OOB) error for internal validation to reduce over-reliance on the training set (Liu C. et al., 2023; Alderden et al., 2018). Candidate targets were defined as genes that simultaneously satisfied a non-zero LASSO coefficient and an RF importance ranking above the median of all candidate genes. This multi-algorithm consensus rule aims to reduce the risk of false positives caused by the overfitting of a single algorithm.

Regarding the evaluation of candidate gene diagnostic efficacy, traditional artificial neural network (ANN) methods are highly prone to overfitting with small sample sizes (n = 10), resulting in inflated AUC estimates that fail to reflect the true generalization ability of the model (Beck, 2018). Therefore, this study abandons the use of ANN for evaluation and instead adopts leave-one-out cross-validation (LOOCV). By leaving out one sample as the validation set each time and using the remaining nine samples as the training set, the process is repeated 10 times until each sample has been independently predicted once. This ensures that the predicted probability for each sample comes from a model that has never seen that sample, thus providing an unbiased estimate of the model’s generalization ability. The “pROC” package is used to calculate the AUC value based on the independent predicted probabilities generated by LOOCV, and the ROC curve is plotted to evaluate the diagnostic efficacy of the gene (Robin et al., 2011). Ninety-five percent confidence intervals (CIs) for the LOOCV-AUC values were estimated by bootstrap resampling of the out-of-fold predictions (B = 2,000; random seed = 12,345) using the “pROC” package. Significance testing for differences in expression between groups is performed using the “stats” package.

#### 2.1.4 Gene set enrichment analysis and molecular regulatory networks

To explore the biological pathways associated with the expression level of the candidate gene WNT4, this study employed Gene Set Enrichment Analysis (GSEA). Samples were divided into high-expression and low-expression groups based on the median WNT4 expression level, and enrichment differences between the two groups were tested using the KEGG pathway gene set (c2. cp.kegg.v7.3. symbols) (P < 0.05) (C et al., 2024). It should be noted that the GSEA results reflect the correlation between WNT4 expression levels and pathway activity, rather than the pathways in which WNT4 is directly involved.

Meanwhile, to preliminarily explore the potential regulatory mechanisms of WNT4, this study constructed a TFs-mRNAs-miRNAs-lncRNAs regulatory network using tools and databases such as TRRUST, PITA, microT, TargetScan, miRNet, starBase, LncBase v3, and RPISeq, to identify core loops potentially involved in the regulation of WNT4 expression. Among these, RPISeq was used to predict lncRNA-TF interactions, which output interaction reliability scores based on random forest (RF) and support vector machine (SVM) classifiers (with RF ≥ 0.90 and SVM ≥0.90 defined as high-confidence predictions). It should be emphasized that this network is entirely based on database predictions, and all regulatory relationships have not been experimentally validated; therefore, it should be regarded as a hypothesis-generating tool rather than a confirmed regulatory mechanism.

#### 2.1.5 Targeted drug search

To pursue existing drug utilization opportunities for candidate targets, drug-gene interactions were explored using DrugBank (https://go.drugbank.com) to identify drugs associated with key targets (Knox et al., 2024).

### 2.2 Targeted drug discovery

#### 2.2.1 Protein target structure modeling and assessment

Given that there are no known targeted drugs for the candidate targets in DrugBank, this study further conducted computational drug discovery work based on natural products and marine products. The target proteins in this study were human WNT4 proteins, corresponding to the results of Section 1.1.

The study obtained the amino acid sequence of the target protein from UniProt (https://www.uniprot.org/), used AlphaFold for structure prediction, and used PyMOL to complete the visualization checking and preprocessing (UniProt Consortium, 2023). The average pLDDT score of the AlphaFold predicted structure is 93.12, indicating high confidence in the overall structural prediction. Per-residue pLDDT values at the predicted binding pocket were extracted from the AlphaFold output for structure-based screening confidence assessment.

According to the literature, the known lipid modification site at the thumb tip of the WNT family and its loop region constitute the key functional interface for binding to Frizzled receptors (Janda et al., 2012). The lipid modification site of the WNT4 protein is located at Ser212 (the thumb domain terminus), providing a structural reference for subsequent binding pocket prediction (Zhang et al., 2021).

It should be noted that currently there is no experimentally resolved human WNT4 crystal or cryo-EM structure. The structure used in this study is a computational prediction model, and its binding pocket along with the binding modes of candidate compounds require further experimental validation.

#### 2.2.2 Natural and marine product ligand library construction

Small molecule ligands for this study were obtained from the Collection of Open Natural products (COCONUT) (https://coconut.naturalproducts.net/) and the Comprehensive Marine Natural Products Database (CMNPD) (https://cmnpd.org/) (Sorokina et al., 2021; Lyu et al., 2021). From these, the study downloaded a dataset of over 700,000 compounds and applied the Lipinski five rules and the Quantitative Estimate of Druglikeness (QED) for initial filtering. The filtering criteria were: violation of no more than one of the RO5 and QED score more than 0.5. After screening, 37,280 and 47,450 candidate compounds were obtained from COCONUT and CMNPD, respectively, for subsequent virtual screening.

#### 2.2.3 Activity pocket prediction and virtual screening

In this study, an automated screening process from binding site identification to molecular docking was constructed. First, the Unified Spatial-Temporal Enhanced model (Uni-STE; implemented via the UniSite framework, GitHub: https://github.com/quanlin-wu/unisite, pre-trained model v1) was deployed to realize the automatic identification of protein binding pockets. The target protein preprocessing file was transformed into spatial map data containing atomic coordinates and chemical features, and the residue spatial features were extracted and inferred by the model’s physically-aware evolutionary operator to compute the protein surface binding probability score, and spatial clustering of high-scoring regions to identify potential pocket coordinates.

Based on this, the study used DiffDock v1.1 (DiffDock-L; GitHub: https://github.com/gcorso/DiffDock), a molecular docking tool based on deep learning diffusion modeling, to perform high-throughput virtual screening to efficiently predict the binding posture and conformational space of small molecules and targets (Corso et al., 2023). Inference was run with 15 diffusion steps and five sampled poses per ligand (samples_per_complex = 5), using the default expbeta sigma schedule; the rank-1 pose for each ligand was retained for downstream ranking. According to the official interpretation guidelines of the DiffDock developers, a confidence score >0 is considered high confidence, −1.5 to 0 as moderate confidence, and < −1.5 as low confidence. It should be noted that these cutoffs are heuristic guidelines suggested by the developers rather than formally validated benchmarks derived from redocking against positive controls. This study adopts >0 and ≥ −1.5 as screening thresholds, and this threshold strategy is designed to improve screening efficiency while retaining a certain proportion of candidate compounds.

#### 2.2.4 Molecular binding modes and interaction analysis

Using software such as PyMOL, Autodock vina, and Protein-Ligand Interaction Profiler (PLIP) (https://plip-tool.biotec.tu-dresden.de/) (Salentin et al., 2015), the study systematically analyzed hydrogen bonding between proteins and ligands, hydrophobic interactions, π-stacking, salt bridges and other non-covalent interactions to clarify the binding mode and key residues.

#### 2.2.5 Molecular dynamics simulations

100-ns all-atom molecular dynamics simulations were performed using the GROMACS (version: 2025.1) software package (Abraham et al., 2015). System construction: Starting with the optimal conformation, the protein was described by the AMBER99SB-ILDN force field; topological parameters of the ligand were generated by ACPYPE (https://bio2byte.be/), with charges fitted using the Bond Charge Correction (BCC) method. The complex was placed at the center of a cubic box with a minimum distance of 10 Å between the box wall and the protein surface, solvated with the TIP3P water model, and neutralized by adding Na^+^ or Cl^−^ ions. Energy minimization and equilibration: Energy minimization was performed using the steepest descent method (convergence threshold: 1000 kJ/(mol·nm)), followed sequentially by a 50-ps constant-volume (NVT) heating (0–298.15 K) and a 50-ps constant-temperature constant-pressure (NPT) equilibration (1 bar, 298.15 K), with positional restraints of 1000 kJ/(mol·nm (Saltiel, 2021)) applied to protein heavy atoms. Production simulation: After removing the positional restraints, a 100-ns unrestrained simulation was performed with a time step of 2 fs and LINCS algorithm applied to constrain bond lengths. Temperature and pressure were coupled via the v-rescale thermostat and Parrinello-Rahman pressure coupling, respectively. The van der Waals cutoff was set to 1.0 nm (10 Å), and electrostatic interactions were calculated using the Particle Mesh Ewald (PME) method. Trajectories were saved every 10 ps.

#### 2.2.6 Free energy calculations

To quantitatively assess the binding affinity of the ligand to the protein, the MM-GBSA method was used to calculate the binding free energy (Hou et al., 2011). From the last 10% of the 100-ns simulation trajectory, one frame was extracted every 10 frames using the GROMACS built-in tool gmx trjconv, yielding a total of 100 frames for the calculation. The entire process was implemented through gmx_MMPBSA. The energy calculation was based on the AMBER99SB-ILDN force field, the gas-phase energy was calculated by the sander module, the solvation energy was estimated by the GB model (igb = 5, i.e., the GB-OBC1 model), and the non-polar solvation energy was calculated by the solvent accessible surface area (SASA) model. In addition, energy decomposition analysis was performed to decompose the total binding energy to each amino acid residue to identify hotspot residues that maintain the stable binding of the complex.

The binding energy values in this study should primarily be used for the relative ranking of candidate compounds, rather than for the precise determination of absolute binding affinity. It should be noted that the MM-GBSA calculations in this study are based on conformations extracted from a single MD trajectory, without multiple independent replicate simulations; additionally, the conformational entropy change (-TΔS) term was not calculated. Therefore, the reported ΔTOTAL represents an estimate of the binding enthalpy change (ΔH), rather than the complete binding free energy (ΔG). This approach may lead to a systematic overestimation of binding affinity due to the neglect of entropy contributions and incomplete sampling from a single trajectory.

#### 2.2.7 Density functional theorycalculations

Density Functional Theory (DFT) calculations were carried out to investigate the molecular properties and reactivity of the candidate compounds at the electronic structure level. All quantum chemical calculations were performed using ORCA 6.1. The unconstrained geometry optimization of the molecules was conducted at the RI-B3LYP-D3(BJ)/def2-TZVP level of theory under gas-phase conditions, and vibrational frequency analysis was performed at the same level to confirm that the optimized structures were minima on the potential energy surface (no imaginary frequencies). Based on the optimized structures, the frontier molecular orbital (HOMO/LUMO) distributions and orbital energy gaps were calculated. Visualization of the calculated results was accomplished using Multiwfn 3.8_dev in conjunction with VMD.

The DFT results in this study should be understood as a qualitative description of the intrinsic electronic properties of the candidate compounds, rather than an accurate simulation of their electronic behavior in the bound state. They are primarily used to aid in understanding the differences in electronic structure among various compounds. It should be noted that the DFT calculations in this study were performed under gas-phase conditions, without the introduction of implicit solvent models (such as PCM or SMD). The gas-phase environment differs from the actual protein binding pocket (which has low dielectric constants and varying local polarities), and the electronic structure in the gas phase may differ from the actual electronic state in a solvated or protein environment.

## 3 Results

### 3.1 Disease target discovery

#### 3.1.1 Candidate gene set screening

WGCNA was used to identify gene modules associated with phenotypic differences. In BR vs. Pre, the soft threshold power β was set to 4 (Figure 2A), and the dynamic tree cut algorithm identified 23 gene modules. Among these, the black module (r = 0.83, P = 6.8e-6) was significantly correlated with the trait (Figures 2B,E), containing 319 module genes (Supplementary Table S1). In RE vs. BR, β was set to 5 (Figure 2C), and 23 gene modules were similarly identified, of which the purple module (r = 0.74, P = 2.1e-4) and red module (r = 0.73, P = 2.2e-4) were significantly correlated with the trait (Figures 2D,F,G), containing 237 and 417 module genes, respectively (Supplementary Table S1).

**FIGURE 2:**
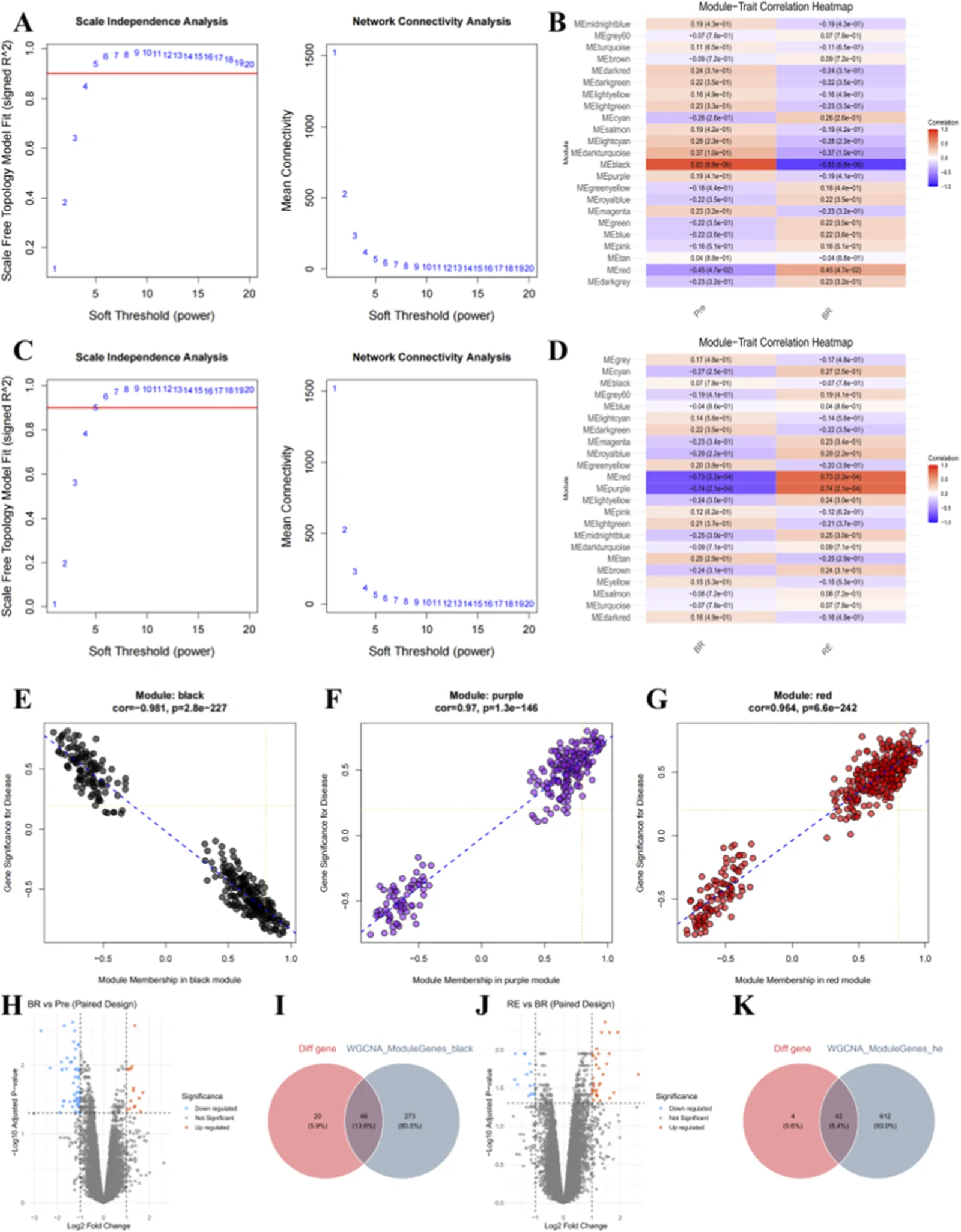
Screening based on WGCNA and differentially expressed genes (DEGs). **(A)** Soft threshold power β selection for BR vs. Pre. **(B)** Module–trait correlation heatmap for BR vs. Pre. **(C)** Soft threshold power β selection for RE vs. BR. **(D)** Module–trait correlation heatmap for RE vs. BR. **(E)** Scatterplot of gene significance vs. module membership in the black module (BR vs. Pre). **(F)** Scatterplot of gene significance vs. module membership in the purple module (RE vs. BR). **(G)** Scatterplot of gene significance vs. module membership in the red module (RE vs. BR). **(H)** Volcano plot of DEGs in BR vs. Pre. **(I)** Venn diagram showing the overlap between WGCNA key modules and DEGs in BR vs. Pre. **(J)** Volcano plot of DEGs in RE vs. BR. **(K)** Venn diagram showing the overlap between WGCNA key modules and DEGs in RE vs. BR.

Meanwhile, GEO2R was used for intergroup normalization and differential expression analysis. In BR vs. Pre, 66 DEGs were identified (Figure 2H); in RE vs. BR, 46 DEGs were identified (Figure 2J). The intersection of the above differential modules with DEGs yielded 46 and 42 candidate genes for the two phases, respectively (Figures 2I,K; Supplementary Table S2).

#### 3.1.2 Screening of candidate genes and evaluation of diagnostic efficacy

Multiple machine learning algorithms were further applied to screen the candidate gene set. In BR vs. Pre, LASSO regression identified eight potential biomarkers (AQP7, BDH1, GPD2, KLF15, LOC728978, MOGAT3, SDHC, WNT4) (Figure 3A); RF screened 11 core genes (BDH1, AQP7, GPD2, PLIN5, ACSS1, SDHC, MPP6, LOC728978, SLC26A9, ACOT11, WNT4) based on the median MeanDecreaseGini value (Figure 3B). In RE vs. BR, eight potential biomarkers were identified (EXTL1, ADAMTS9-AS2, CCND1, CTGF, FOXM1, IGFBP2, SPRY4, WNT4) (Figure 3C); RF screened 11 core genes (ADAMTS9-AS2, IGFBP2, CTGF, DCX, SPRY4, FOXM1, WNT4, SPTBN5, EXTL1, NEDD9, TMEM163) (Figure 3D).

**FIGURE 3:**
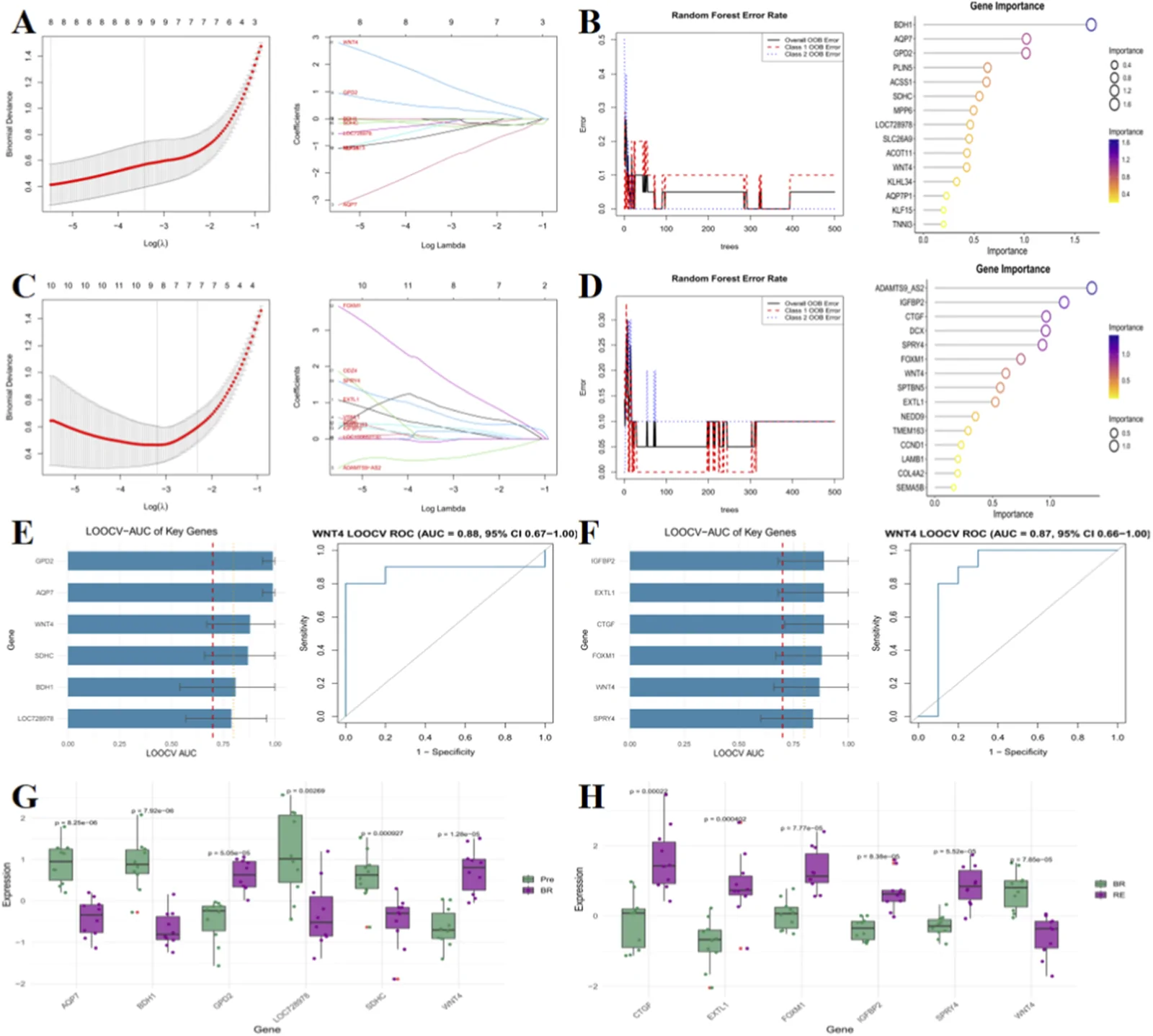
Candidate gene screening and validation based on machine learning. **(A)** LASSO regression for BR vs. Pre. **(B)** Random forest analysis for BR vs. Pre. **(C)** LASSO regression for RE vs. BR. **(D)** Random forest analysis for RE vs. BR. **(E)** LOOCV-AUC values of candidate genes for BR vs. Pre and the ROC curve of WNT4. **(F)** LOOCV-AUC values of candidate genes for RE vs. BR and the ROC curve of WNT4. **(G)** Expression differences of candidate genes between groups for BR vs. Pre. **(H)** Expression differences of candidate genes between groups for RE vs. BR.

After multi-algorithm overlap comparison, the following candidate targets were identified: AQP7, BDH1, GPD2, LOC728978, SDHC, WNT4 for BR vs. Pre; EXTL1, CTGF, FOXM1, IGFBP2, SPRY4 and WNT4 for RE vs. BR, and WNT4 was the only gene selected in both stages.

To more robustly evaluate the independent diagnostic efficacy of each candidate gene, the study employed LOOCV to calculate the AUC for each gene. Under this framework, the predicted value of each sample comes from a model that has never seen that sample, thereby providing an unbiased estimate of generalization ability. In the BR vs. Pre comparison (Figure 3E), the LOOCV-AUC values of the six candidate genes, ranked from high to low, were as follows: AQP7 (0.99, 95% CI: 0.94–1.00), GPD2 (0.99, 95% CI: 0.94–1.00), WNT4 (0.88, 95% CI: 0.67–1.00), SDHC (0.87, 95% CI: 0.66–1.00), BDH1 (0.81, 95% CI: 0.54–1.00), and LOC728978 (0.79, 95% CI: 0.57–0.96). In the RE vs. BR comparison (Figure 3F), the LOOCV-AUC values of the six candidate genes were: EXTL1 (0.89, 95% CI: 0.68–1.00), CTGF (0.89, 95% CI: 0.71–1.00), IGFBP2 (0.89, 95% CI: 0.68–1.00), FOXM1 (0.88, 95% CI: 0.67–1.00), WNT4 (0.87, 95% CI: 0.66–1.00), and SPRY4 (0.84, 95% CI: 0.60–1.00). Notably, WNT4 showed highly consistent LOOCV-AUC values across the two independent comparison stages (BR vs. Pre: 0.88; RE vs. BR: 0.87), and it was the only candidate gene that demonstrated robust performance in both stages.

Further analysis of expression differences revealed that all candidate genes showed significant differences between groups (Figures 3G,H). Notably, WNT4 expression during the Pre-BR-RE process exhibits a bidirectional pattern of initial increase followed by a decrease, suggesting its potential involvement in the dynamic regulation of skeletal muscle IS.

#### 3.1.3 Gene set enrichment analysis and molecular regulatory network

To explore the biological pathways associated with WNT4 expression levels, GSEA was performed on skeletal muscle samples from BR vs. Pre and RE vs. BR, respectively (Supplementary Table S3). The samples were divided into high-expression and low-expression groups based on the median WNT4 expression level, and the enrichment differences of KEGG pathway gene sets between the two groups were examined. The results showed that the pathways positively correlated with high WNT4 expression were highly overlapping under both interventions, mainly including cardiac muscle contraction, oxidative phosphorylation, and Alzheimer’s disease. Only three pathways were specifically differentially enriched between the different intervention factors: the hedgehog signaling pathway was specifically associated with the bed rest factor, while purine metabolism and endometrial cancer were specifically associated with the exercise retraining factor; among them, only purine metabolism showed a positive correlation between expression and phenotypic changes. It should be noted that the GSEA results reflect the correlation between WNT4 expression levels and pathway activity, rather than the pathways in which WNT4 is directly involved; furthermore, the enrichment of the “endometrial cancer” pathway may arise from cross-overlap within KEGG gene sets and may have no direct biological significance in the context of skeletal muscle in this study.

Further, a TFs-mRNAs-miRNAs-lncRNAs predictive regulatory network was constructed around WNT4. A total of 60 regulatory axes were obtained, and 28 potential circular regulatory loops were screened. Among these, lncRNAs-TFs prediction confidence was high for the EGR1 - WNT4 - hsa-miR-1238-3p - LINC00969 and EGR1 - WNT4 - hsa-miR-1238-3p - PRKCQ-AS1 circular regulatory loops (RF ≥ 0.90, SVM ≥0.90) (Figures 4A,B).

**FIGURE 4:**
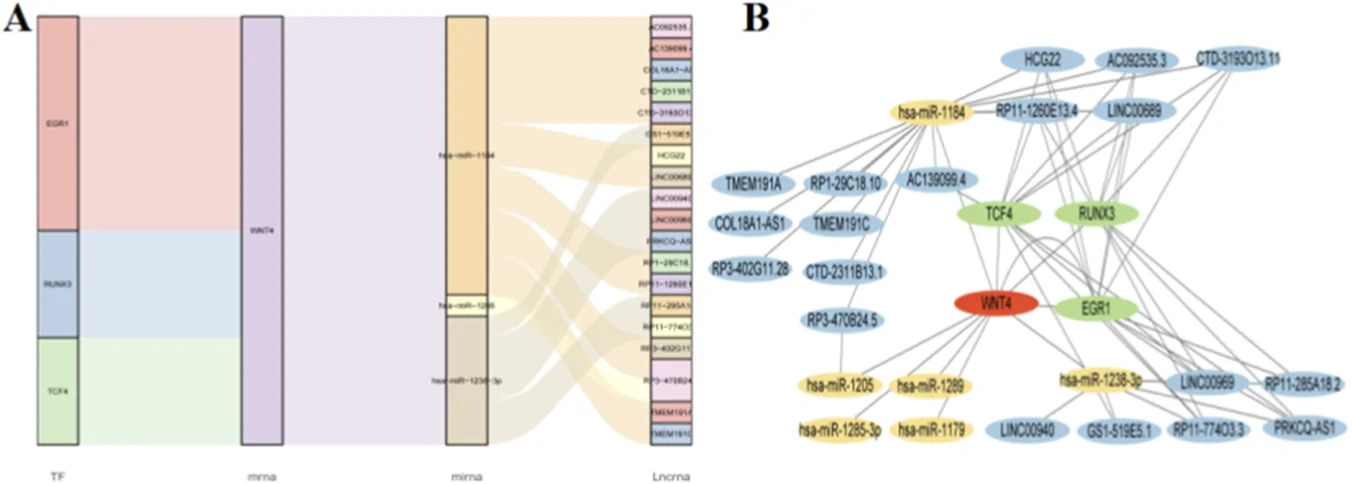
TFs-mRNAs-miRNAs-lncRNAs regulatory network centered on WNT4. **(A)** The alluvial diagram of the TFs-mRNAs-miRNAs-lncRNAs network. **(B)** Circular regulatory signaling pathway.

#### 3.1.4 Drug-gene interaction analysis

A search of the DrugBank database for drug-gene interactions with WNT4 revealed that there are currently no approved therapeutic drugs directly targeting WNT4. This result indicates that the development of targeted drugs for WNT4 remains a blank field, further supporting the value of subsequent computational-based screening of candidate compounds.

### 3.2 Drug discovery

#### 3.2.1 WNT4 protein structure modeling and assessment

The amino acid sequence of WNT4 (accession number: P56705) was retrieved from the UniProt database, and three-dimensional structure prediction was performed using AlphaFold. The final predicted structure achieved an average pLDDT score of 93.12, indicating high prediction confidence. At the predicted binding pocket (near CYS208/GLY210/SER214), the per-residue pLDDT values were 92.38, 84.44, and 81.06. The PAE matrix further validated the reasonableness of the relative spatial positions between structural domains.

#### 3.2.2 Construction of natural product and marine product ligand libraries

Over 700,000 small molecules were downloaded and subjected to initial filtering using RO5 and QED. Ultimately, 37,280 and 47,450 candidate compounds were obtained from COCONUT and CMNPD, respectively.

#### 3.2.3 Active pocket prediction and virtual screening

First, Uni-STE was deployed to predict potential binding pockets on the WNT4 protein. Subsequently, DiffDock was used to perform deep learning-based molecular docking of the filtered ligands against the target protein. During the DiffDock inference process 5,411 compounds were automatically skipped due to failures in internal conformer generation or model inference, ultimately resulting in 79,319 successfully docked molecules. The Rank one optimal pose for each molecule was selected and ranked by confidence score (Supplementary Table S4). The results showed that 181 compounds with confidence score >0 were classified as high-confidence candidates, while 5,740 compounds with confidence score ≥ −1.5 were classified as medium-confidence candidates.

#### 3.2.4 Molecular binding mode and interaction analysis

To elucidate the predicted binding modes of the candidate compounds with WNT4, the top three candidate compounds ranked by DiffDock confidence score (CMNPD4923, CMNPD27319, CMNPD30941) were selected, and non-covalent interactions were evaluated using PLIP. All three molecules stably bound to the same active region of WNT4 (near CYS208, GLY210, and SER214), which is centered around the known lipid modification site Ser212 (at the end of the thumb domain) of WNT4. The molecules primarily interact with the aforementioned residues through hydrogen bonds, with bond distances concentrated between 1.70 and 2.42 Å (Figure 5; Table 1). Among them, CMNPD27319 formed multiple strong hydrogen bonds with GLY210 and SER214, suggesting that it may possess relatively strong binding potential.

**FIGURE 5:**
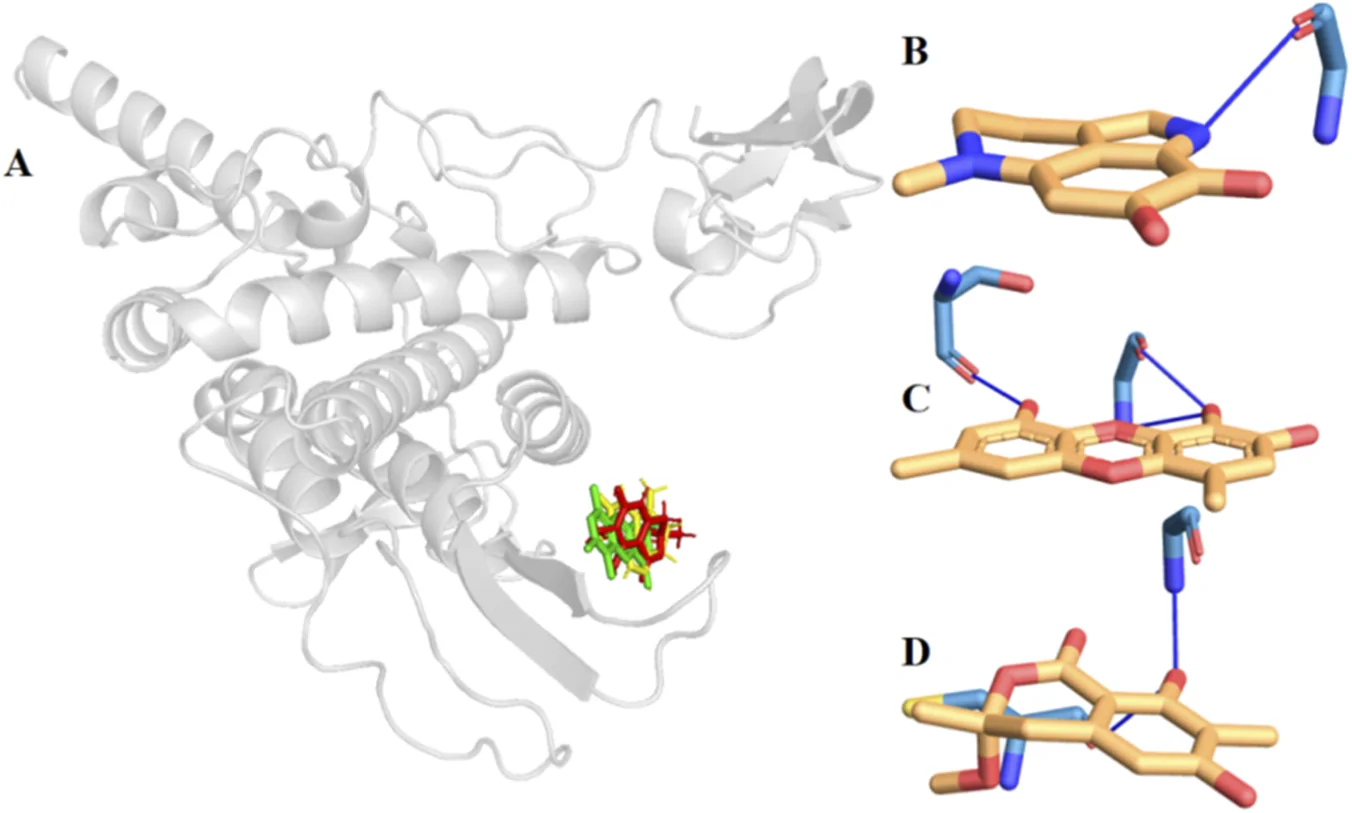
Binding mode analysis of the top three candidate compounds with WNT4. Ligands and key interacting residues are displayed as sticks, and hydrogen bonds are indicated by blue solid lines. **(A)** Overall binding modes of the three complexes. **(B)** CMNPD4923–WNT4 complex. **(C)** CMNPD27319–WNT4 complex. **(D)** CMNPD30941–WNT4 complex.

**TABLE 1:** Hydrogen bond interactions between the top three candidate compounds and WNT4 key residues identified by PLIP.

	Residue	AA	Distance H-A	Ligand Atom D-A	Donor Angle	Donor Atom	Acceptor Atom
CMNPD4923	210A	GLY	2.29	2.91	118.88	1 [Npl]	1640 [O2]
CMNPD27319	210A	GLY	2.32	3.19	146.74	1631 [Nam]	19 [O3]
	210A	GLY	1.75	2.45	128.97	19 [O3]	1634 [O2]
	214A	SER	1.70	2.58	154.36	17 [O3]	1655 [O2]
CMNPD30941	208A	CYS	2.42	3.17	133.65	16 [O2]	1630 [O2]
	210A	GLY	2.28	3.04	132.73	1643 [Nam]	16 [O2]

#### 3.2.5 Molecular dynamics simulations

Molecular dynamics simulations of the top three candidate small molecules (CMNPD4923, CMNPD27319, CMNPD30941) ranked by DiffDock confidence score in complex with WNT4 were performed using GROMACS. Four indicators were primarily analyzed: root mean square deviation (RMSD), root mean square fluctuation (RMSF), solvent accessible surface area (SASA), and radius of gyration (Rg) (Figure 6A).

**FIGURE 6:**
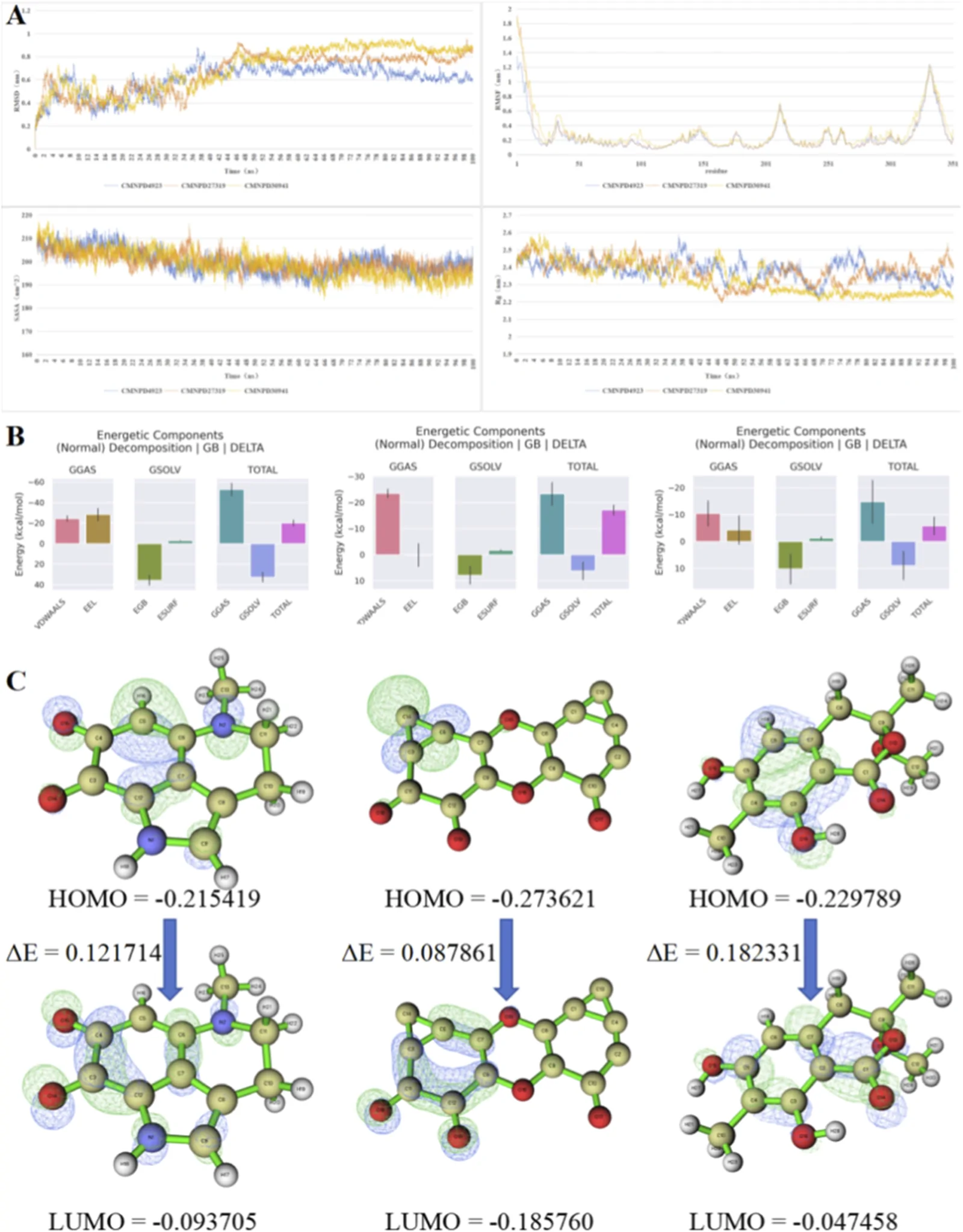
Molecular dynamics simulation and DFT analysis of the top three candidate compound–WNT4 complexes. **(A)** RMSD, RMSF, SASA, and Rg plots from 100 ns MD simulations. **(B)** Binding free energy calculated by MM-GBSA. **(C)** HOMO–LUMO orbital distributions and energy gaps from DFT calculations.

RMSD analysis showed noticeable initial system fluctuations, attributable to the optimization of the initial DiffDock poses within the force field. During the 50–100 ns period, all three complexes reached equilibrium, with mean RMSD ±SD values of 0.6659 ± 0.0480, 0.7855 ± 0.0310, and 0.8639 ± 0.0492 nm, respectively, indicating that each system achieved a relatively stable equilibrium state. RMSF analysis revealed that the proportions of highly fluctuating residues (>0.3 nm) in the three systems were 18.5%, 22.5%, and 30.5%, respectively, indicating that ligand binding did not induce drastic changes in the overall rigidity of the protein. SASA analysis showed mean ± SD values of 199.6571 ± 4.9840, 199.7644 ± 3.8571, and 198.7523 ± 5.7994 nm^2^ for the three systems; concurrently, Rg analysis showed mean ± SD values of 2.3814 ± 0.0572, 2.3767 ± 0.0696, and 2.3308 ± 0.0911 nm. The small fluctuations in SASA and Rg values do not support the possibility of ligand binding-induced protein unfolding.

The above results indicate that the three candidate compounds maintain relatively stable complex conformations upon binding to WNT4, providing preliminary structural support for their potential binding modes.

#### 3.2.6 Binding free energy calculations

This study employed the MM-GBSA method to calculate the binding free energies of CMNPD4923, CMNPD27319, and CMNPD30941 with WNT4 complexes to further evaluate the thermodynamic characteristics of its binding pattern. The results showed that the total binding energies (ΔTOTAL) for the three systems were −20.08 ± 2.97, −17.13 ± 1.87, and −5.80 ± 3.32 kcal/mol, respectively (Figure 6B).

In terms of energy component characteristics, ΔGGAS was the core driving force for binding, with ΔVDWAALS being the dominant contributor; ΔEEL also provided additional favorable contributions to the binding in the CMNPD4923 system. In contrast, within ΔGSOLV, ΔEGB exhibited significantly positive values overall, imposing a substantial desolvation penalty and representing the primary unfavorable contributor to binding (Supplementary Table S5).

Regarding residue energy contributions, specific residues (GLY210, GLY213, SER214) consistently showed consistent binding energy contributions across all three systems. These residues primarily provided strong van der Waals and electrostatic interactions, while partially offsetting the overall desolvation penalty at the polar solvation level, thereby serving as common key sites maintaining system stability (Supplementary Table S6). Notably, residue contribution analysis also revealed that LYS207 and the ligand provided major favorable contributions during the binding process of the CMNPD27319 system.

Overall, the ΔTOTAL values of CMNPD4923 and CMNPD27319 are significantly negative, suggesting their relatively strong binding potential; the ΔTOTAL value of CMNPD30941 is notably weaker, indicating its binding capacity is relatively limited. The energy differences among the three molecules suggest that the DiffDock confidence score and the MM-GBSA binding free energy are not entirely consistent.

#### 3.2.7 DFT calculations of small molecule drugs

To further explore the electronic structure characteristics of the candidate compounds, DFT calculations were performed, analyzing the highest occupied molecular orbital (HOMO), the lowest unoccupied molecular orbital (LUMO), and their energy gap (ΔE). The results showed that the ΔE values for CMNPD4923, CMNPD27319, and CMNPD30941 were 0.122, 0.088, and 0.182 a. u., respectively (Figure 6C). In terms of spatial distribution, the HOMO orbitals of the three molecules were mainly localized in electron-rich regions such as aromatic rings and methylene (-CH-), while the LUMO orbitals were primarily distributed in electron-deficient regions including carbonyl (C=O), imine (-NH-), and hydroxyl (-OH) groups. These distribution characteristics suggest that these molecules possess active electron cloud distributions and potential charge transfer features.

## 4 Discussion

IR is a common pathological basis of multiple chronic non-communicable diseases, and physical activity is considered one of the effective non-pharmacological strategies for improving IS. However, the core molecular switch mediating exercise-regulated skeletal muscle IS and its dynamic regulatory mechanisms remain unclear. This study introduced a bidirectional skeletal muscle dataset based on a “bed rest-induced IR and resistance exercise-reversed IR” model, integrated multi-omics and computational chemistry strategies, preliminarily identified WNT4 as a candidate target for the dynamic regulation of skeletal muscle IS, and established a potential lead compound computational pipeline for the discovery of candidate compounds for its translational application.

WNT4 was jointly screened by WGCNA and differential expression analysis in both the BR vs. Pre and RE vs. BR phases, and demonstrated good discriminatory performance through multi-algorithm integrated validation, making it the only candidate gene repeatedly identified across two phases and multiple methods. In terms of dynamic expression, WNT4 exhibited a bidirectional pattern of “upregulation during BR and downregulation following RE”. This temporal characteristic suggests a tight responsive coupling between its expression level and skeletal muscle metabolic status, rather than a unidirectional injury-related change.

Multiple lines of evidence support that, during the early stage of bed rest-induced disuse, WNT4 may plays the role of an “endogenous compensatory factor” in skeletal muscle. First, WNT4 is an inhibitor of the canonical Wnt pathway, capable of completely blocking WNT3a-stimulated cell growth and insulin secretion, thereby preventing its aberrant fluctuations under metabolic stress (Bowen et al., 2016). Second, WNT4 is a key factor for maintaining muscle stem cell quiescence; its overexpression under injury conditions delays satellite cell activation, favoring the preservation of skeletal muscle regenerative potential in response to metabolic stress (Eliazer et al., 2019). Combined with the GSEA results of this study, among the top 30 pathways positively correlated with WNT4 expression, the majority were related to energy metabolism, encompassing core metabolic nodes such as oxidative phosphorylation, the TCA cycle, fatty acid metabolism, pyruvate metabolism, branched-chain amino acid degradation, and the PPAR signaling pathway. This highly focused functional landscape suggests that the functional output of WNT4 in skeletal muscle may not be a multidirectional injury response, but rather directed toward energy homeostasis regulation. Based on the above correlations and literature evidence, we hypothesize that the reactive elevation of WNT4 may represent a compensatory protective mechanism by which skeletal muscle, when faced with mechanical deprivation, attempts to locally stabilize the mitochondrial network and maintain internal environmental homeostasis.

However, we further speculate that when bed rest stress persists, this compensatory mechanism may undergo a qualitative transformation. Locally within skeletal muscle, sustained high expression of WNT4 leads to excessive inhibition of the canonical Wnt/β-catenin pathway, depriving this pathway of its long-term support for skeletal muscle metabolism and consequently affecting its regulation of skeletal muscle insulin sensitivity. The possible related mechanisms involve the exacerbation of intramyocellular lipid deposition and the functional inhibition of key insulin signaling nodes such as AMPK and IRS-1 (Zhou et al., 2012; Karczewska-Kupczewska et al., 2016; Tian et al., 2021). This inference is consistent with previous studies demonstrating that skeletal muscle-specific β-catenin knockout significantly impairs insulin-stimulated glucose uptake (Masson et al., 2023). More critically, this risk is not confined to skeletal muscle. WNT4 is a secretable myokine; once locally over-accumulated, it can enter the circulation and exert systemic effects (Zhang et al., 2021). The above hypothesis needs to be further validated through functional experiments such as WNT4 knockdown/overexpression.

Under pathological conditions such as T2DM, WNT4 is significantly upregulated (Kozinski et al., 2016; Kurita et al., 2019). As a biphasic initiator of both canonical (β-catenin-dependent) and non-canonical (PCP and Wnt/Ca^2+^) pathways, excess WNT4 can inhibit β-catenin activity by activating the non-canonical PCP/JNK pathway, and completely block the WNT3a-mediated pro-proliferation and pro-insulin secretion effects (Zhang et al., 2021; Mentink et al., 2018). Therefore, the massive secretion of WNT4 by skeletal muscle under long-term metabolic stress may pose a potential endocrine threat to islet function. Previous studies have revealed that the secretion profiles of the canonical Wnt activator (WNT3a) and the Wnt inhibitor (WNT4) are distinctly different between the prediabetic and diabetic stages—WNT3a increases in the prediabetic stage, whereas WNT4 elevates in the diabetic stage (Kozinski et al., 2016). The upregulation of WNT4 observed in this study may reflect a compensatory regulation initiated by the body to cope with metabolic disorders, but its sustained local overexpression may trigger a cascade of decompensation risks, which still requires further investigation.

The phase-specific functional transition of WNT4 is inextricably linked to the drive of its underlying complex molecular network. The TFs-mRNAs-miRNAs-lncRNAs predictive regulatory network constructed in this study suggests that the upstream core transcription factor EGR1 initiates WNT4 transcription in response to metabolic pressure, while the ceRNA network formed by hsa-miR-1238-3p in combination with LINC00969 and PRKCQ-AS1 finely tunes WNT4 expression at the post-transcriptional level. It should be emphasized that this network is entirely based on database predictions, and all regulatory relationships have not been experimentally validated; therefore, it should be regarded as a hypothesis-generating tool rather than a confirmed regulatory mechanism. If this predicted network is experimentally validated, it will indicate that WNT4 expression is not a simple “on-off” mode, but rather a system endowed with dynamic buffering and adaptive regulation.

The complexity of WNT4 regulation highlights the dilemmas in its targeted drug development. A search of DrugBank confirmed that there are currently no mature clinical drugs directly targeting WNT4. Existing intervention strategies are mostly indirect, such as the exogenous supplementation of recombinant WNT4 protein or adeno-associated virus-mediated WNT4 overexpression; however, these strategies fail to achieve precise spatiotemporal control over WNT4 expression levels and functional states (Xu et al., 2022; Cheng et al., 2024). More critically, the function of WNT4 is highly dependent on specific tissue and cellular contexts: WNT4 upregulation can enhance the osteogenic differentiation capacity of bone marrow mesenchymal stem cells, but it can also exacerbate endothelial cell dysfunction and drive the progression of atherosclerosis (Gao et al., 2024; Schwartze et al., 2025). This tissue specificity and functional dichotomy mean that, under the specific pathological background of skeletal muscle IR, existing indirect intervention strategies not only struggle to ensure the precise replication of efficacy but may also trigger unpredictable systemic side effects due to inter-tissue functional differences. Furthermore, the candidate compounds screened in this study were obtained by docking with the WNT4 protein itself, without considering the tissue background or receptor complex environment. Whether they possess skeletal muscle selectivity cannot be inferred from the computational data at present. In the future, if skeletal muscle-specific intervention is required, strategies such as skeletal muscle-targeted delivery systems, co-receptor/microenvironment selective regulation, or prodrug design could be explored, but all require subsequent experimental validation.

Simultaneously, traditional drug development strategies directly targeting the Wnt pathway also face bottlenecks. The WNT ligand family consists of 19 secreted glycoproteins, involving a variety of Frizzled receptors, co-receptors, and signal transducers. Each member possesses differential functions in different tissue contexts; therefore, interventions that agonize or inhibit the Wnt pathway are bound to interfere with normal physiological functions, leading to on-target toxicity (Qin et al., 2023). Consequently, developing drugs that specifically target WNT4 not only helps circumvent the potential risks associated with broad-spectrum Wnt pathway interventions but also facilitates breaking through the tissue and functional limitations of existing WNT4 modulation methods.

In response to the challenges in targeted drug development, this study conducted a preliminary exploration from a computational perspective, constructing a funnel-like pipeline for drug screening and validation to evaluate the potential binding capacity of candidate molecules across multiple dimensions. This study initially screened over 700,000 compounds from the COCONUT and CMNPD databases, locking in a series of candidate compounds.

Traditional docking or structure-based virtual screening faces computational efficiency and structural prediction bottlenecks; therefore, this study introduced DiffDock, a deep docking model. Unlike traditional docking software that relies on fixed conformational sampling, DiffDock learns the conformational distribution of ligand-protein binding from data, avoiding tedious conformational searches and thereby significantly improving computational efficiency (Corso et al., 2023; Vu et al., 2025). Its advantages are even more pronounced for systems with highly flexible ligands or significant induced-fit effects.

It should be noted that the screening results of the aforementioned candidate compounds are based on computational predictions and face several fundamental cognitive limitations. First, the protein structure of WNT4 is predicted by AlphaFold, and no experimentally resolved human WNT4 crystal or cryo-EM structure is currently available. Second, the binding pocket used in this study was predicted *de novo* by the Uni-STE algorithm. Although it is located near CYS208, GLY210, and SER214, an area surrounding the known lipid modification site Ser212 (at the tip of the thumb domain) of WNT4, which provides indirect support for the biological relevance of the predicted pocket, the druggability of this pocket and the true binding modes of the screened candidate compounds still require experimental validation. Furthermore, the DiffDock confidence score is an internal reliability indicator of the model’s output pose and is not directly equivalent to experimental binding affinity.

At the specific verification level, the top three compounds ranked by DiffDock confidence score are the only candidates scoring >1.0, providing a clean numerical cutoff; therefore, they were selected as representative candidates. Although traditional force field-based MD and MMGBSA can effectively describe the dynamic behavior and thermodynamic trends of protein-ligand binding, they struggle to directly characterize the electronic nature of intermolecular interactions. Therefore, building upon MD and MMGBSA, this study further introduced DFT, expanding the evaluation perspective from the classical mechanics level to the quantum electronic structure level.

In the MMGBSA results, the binding energy of CMNPD30941 is significantly weaker compared to CMNPD4923 and CMNPD27319, suggesting its lower priority as a candidate compound. This result also indicates that the DiffDock confidence score is not entirely consistent with the MMGBSA binding free energy, and future research may need to integrate multiple scoring strategies to improve screening reliability. Additionally, the MMGBSA calculations in this study were based on a single MD trajectory and did not include conformational entropy contributions, and the DFT calculations were performed under gas-phase conditions rather than in an aqueous or protein-embedded environment. These methodological limitations may lead to deviations in the numerical values, implying that the relative ranking among compounds is more informative than the absolute values.

It is worth mentioning that CMNPD4923, namely, Damirone B, is an alkaloid derived from marine sponges; CMNPD27319, namely, Aspergilol E, is a diaryl ether secondary metabolite sourced from marine Aspergillus spp.; and CMNPD30941, namely, 6,8-dihydroxy-3-,7-dimethyl-isochroman-1-one, is a polysubstituted isochroman-1-one/isocoumarin derivative commonly found in fungi and other natural products (Blunt et al., 2018; Utkina et al., 2021; Wang et al., 2018). The screening results span different marine/microbial sources, and their structural diversity suggests that the DiffDock model did not fall into structural bias, possessing a relatively broad molecular recognition capability within an ultra-large chemical spaces.

In summary, the deep learning-driven computational drug discovery strategy and multi-scale cascaded validation strategy adopted in this study provide preliminary candidate compounds for the WNT4 target, and demonstrate a generalizable methodological framework for computational drug discovery. This multi-scale methodological integration, ranging from classical mechanics to quantum mechanics, makes the evaluation of candidate molecules in this study more comprehensive and in-depth, providing a replicable technical pathway for natural product-based drug discovery. Concurrently, the study incorporated CMNPD, compared to synthetic compounds, marine and microbial natural products have retained their superiority in interacting with biomolecules over a long evolutionary process, and have irreplaceable structural advantages in addressing complex target druggability (Wiese and Imhoff, 2019). DrugBank searches confirm that there are currently no approved drugs targeting WNT4; the candidate compounds obtained from this screening provide a preliminary chemical starting point for subsequent drug development targeting this protein.

This study has several limitations. At the data level, the target screening was based on a single dataset (GSE24215, n = 10), and WNT4 was not cross-validated against a second independent public transcriptomic cohort. Although the multi-algorithm integration strategy and the bidirectional intervention design partially mitigate the risk of overfitting, the diagnostic performance and expression dynamics of WNT4 still need to be validated in larger sample sizes and independent public transcriptomic cohorts. At the computational level, the drug discovery was entirely computational, while the druggability of the pocket awaits experimental validation, and both MM-GBSA and DFT calculations have methodological limitations. The actual binding affinity and functional activity of the obtained candidate molecules with WNT4 need to be confirmed by *in vitro* experiments such as SPR and MST, as well as cellular-level functional validations, while their pharmacokinetic properties and *in vivo* safety remain to be evaluated. At the biological level, the hypotheses regarding WNT4 “endogenous compensation” and “phase-specific functional transition” needs to be verified through WNT4 knockdown/overexpression experiments; the ceRNA regulatory network also requires experimental confirmation. Finally, how to achieve the spatiotemporally targeted delivery of candidate molecules to skeletal muscle, avoiding interference with the normal functions of WNT4 in other tissues, remains a key issue to be resolved.

## 5 Conclusion

This study utilized a bidirectional model of “bed rest-induced IR and exercise retraining-reversed IR” and integrated multi-omics analysis approaches to preliminarily identify WNT4 as the only candidate gene commonly screened in both stages, exhibiting a bidirectional expression pattern of rising first and then declining during the Pre-BR-RE process. Based on the above correlations and documentary evidence, we hypothesize that the upregulation of WNT4 during the bed rest period may constitute an endogenous compensatory protective mechanism, while its long-term maintenance may participate in pathological progression, with its functional output focusing on energy metabolism pathways such as oxidative phosphorylation. In addition, this study constructed a predictive regulatory network centered on EGR1 and the ceRNA network, providing candidate circuits for further exploring the molecular mechanism of the stage-specific functional transition of WNT4. In response to the translational bottleneck of WNT4, this study established a deep learning-driven computational drug discovery strategy and a multi-scale cascaded binding validation strategy, screening a batch of candidate compounds from over 700,000 natural and marine products. In summary, this study proposes a working hypothesis regarding the dynamic regulation of skeletal muscle IR centered on WNT4, and provides candidate compounds and a generalizable computational methodology framework for future drug development targeting WNT4. All the above findings are computational predictions that require further validation in independent cohorts and experiments.

## Data Availability

The original contributions presented in the study are included in the article/Supplementary Material, further inquiries can be directed to the corresponding author.
